# Supplementary material for: Noncanonical function of folate through folate receptor 1 during neural tube formation
Source: Nat Commun. 2024 Feb 22;15:1642. doi: 10.1038/s41467-024-45775-1 (PMC10883926; doi:10.1038/s41467-024-45775-1)

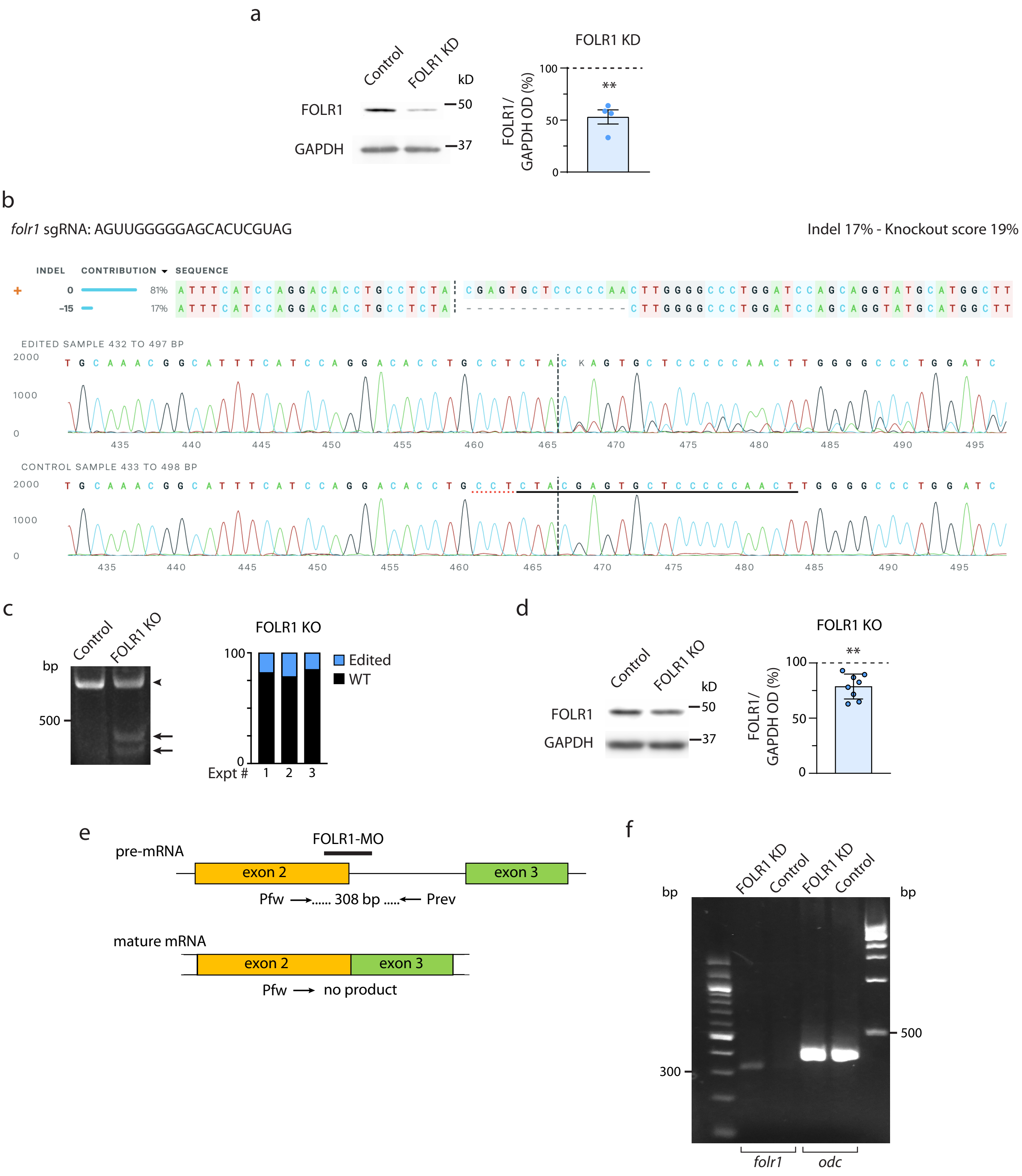

a
Control
FOLR1 KD
kD
FOLR1
GAPDH
50
37
FOLR1 KD
FOLR1/
GAPDH OD (%)
100
50
0
**
b
folr1 sgRNA: AGUUGGGGGAGCACUCGUAG
Indel 17% - Knockout score 19%
INDEL
CONTRIBUTION
SEQUENCE
0
81%
−15
17%
EDITED SAMPLE 432 TO 497 BP
2000
1000
0
CONTROL SAMPLE 433 TO 498 BP
c
Control
FOLR1 KO
bp
500
FOLR1 KO
Edited
WT
100
0
Expt #
1
2
3
d
Control
FOLR1 KO
kD
FOLR1
GAPDH
50
37
FOLR1 KO
FOLR1/
GAPDH OD (%)
100
50
0
**
e
pre-mRNA
FOLR1-MO
exon 2
exon 3
Pfw
308 bp
Prev
mature mRNA
exon 2
exon 3
Pfw
no product
f
FOLR1 KD
Control
FOLR1 KD
Control
bp
bp
500
300
folr1
odc

Supplementary Fig. 1. Folate receptor 1 knockdown and knockout in hiPSCs and *Xenopus laevis* embryos.

a, hiPSCs at 70% confluency were incubated with 2 μM FOLR1-*vivo* morpholino (FOLR1-MO, FOLR1 KD) or Control-*vivo* MO (Control) for 48 h, when collected and processed for Western blot assays. Image is an example of a Western blot assay. Graph shows individual and mean±SD percent of optical density (OD) for FOLR1 protein band normalized with GAPDH and compared to Control (100%, dashed line), $^{**}p<0.01$, one-sample t and Wilcoxon test. **b**-**d**, 60% confluent hiPSCs from 1 well of a 6-well plate were incubated with 1.2 μg FOLR1-targeted gRNA, 6.25 μg Cas9 and lipofectamine CRISPR transfection reagent for 48 h. Four-day old cells were then processed for sequencing, ICE knockout analysis (**b**), detection of genomic cleavage (**c**) and FOLR1 protein level (**d**). **b**, Experimental assessment of CRISPR/Cas9-mediated *FOLR1* gene deletion by ICE knockout analysis. The Cas9 cleavage site is indicated in vertical dashed line. Top, wild-type sequence is marked by + (left, 0). Bottom, Sanger sequencing chromatograms showing edited and control sequences around the region of the guide sequence (horizontal black line). PAM site: dotted red line. **c**, Example of gel showing genomic DNA from control and transfected hiPSCs processed to detect cleavage by CRISPR/Cas9 (lower size bands). Graph shows the proportion of FOLR1 edited cells in transfected hiPSCs in N=3 experiments, exhibiting a 20% *FOLR1* KO. **d**, FOLR1 gRNA/CRISPR/Cas9 transfected (FOLR1 KO) and control hiPSCs at 70% confluency were collected and processed for Western blot assays. Image is an example of Western blot assay. Graph shows individual and mean±SD percent of optical density (OD) for FOLR1 protein band normalized with GAPDH and compared to Control (100%, dashed line), $^{**}p<0.01$, one-sample t and Wilcoxon test. **e**, Schematic of FOLR1 splicing morpholino design against *Xenopus laevis folr1* mRNA. Forward (fw) and reverse (rev) primers (P) used to detect aberrantly spliced mRNA and expected PCR products are indicated under each pre- and mature-mRNA drawings. **f**, mRNA was extracted from neural plate stage control-MO (Control) and FOLR1-MO (FOLR1 KD) injected *Xenopus laevis* embryos and processed for RT-PCR with primers indicated in **e**. Shown is example of PCR product gel with ODC as positive control. Three independent experiments showed similar results.

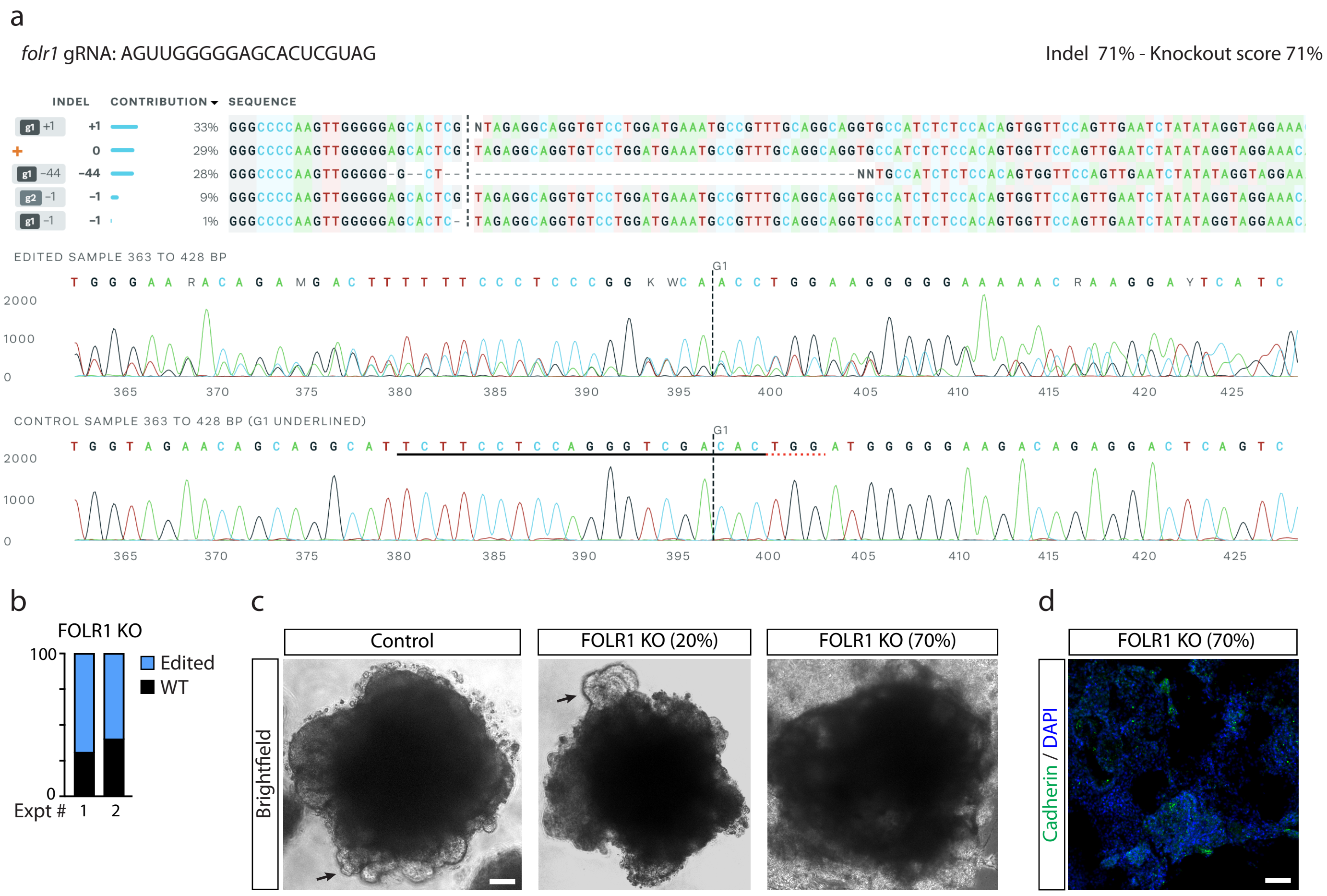
a
folr1 gRNA: AGUUGGGGGAGCACUCGUAG
Indel 71% - Knockout score 71%
INDEL
CONTRIBUTION
SEQUENCE
EDITED SAMPLE 363 TO 428 BP
CONTROL SAMPLE 363 TO 428 BP (G1 UNDERLINED)
b
FOLR1 KO
Edited
WT
Expt #
c
Control
FOLR1 KO (20%)
FOLR1 KO (70%)
Brightfield
d
FOLR1 KO (70%)
Cadherin / DAPI

Supplementary Fig. 2. High score FOLR1 knockout hiPSCs impede development of neural organoids.

Approximately 60% confluent hiPSCs seeded in 6-well plates were incubated with 1.2 μg FOLR1-targeted synthetic gRNA, 6.25 μg Cas9 and lipofectamine CRISPR transfection reagent for 48 h. After sequential dilutions, cells were then seeded on multiwells and allowed to form colonies, which were screened for transformation. Samples were then processed for sequencing, ICE knockout (KO) analysis (**a**,**b**) and morphological assessment (**c**,**d**). **a**, Experimental assessment of CRISPR/Cas9-mediated *FOLR1* gene deletion by ICE KO analysis. The Cas9 cleavage site is indicated in vertical dashed line. Wild-type sequence is marked by + (left, 0). Sanger sequencing chromatograms show edited and control sequences around the region of the guide sequence as underlined with horizontal black line. PAM site is underlined with dotted red line. **b**, Graph shows the proportion of FOLR1 edited cells in transfected hiPSCs in 2 experiments, exhibiting 60-70% *FOLR1* KO. **c**-**d**, Examples of brightfield (**c**) and immunostained (**d**) images of control (wild-type, **c**) 20% (**c**) and 70% FOLR1 KO hiPSC-derived neural organoids (**c**,**d**). Arrows in **c** indicate morphologically distinguishable neural tube-like structures. Scale bars, 100 μm.

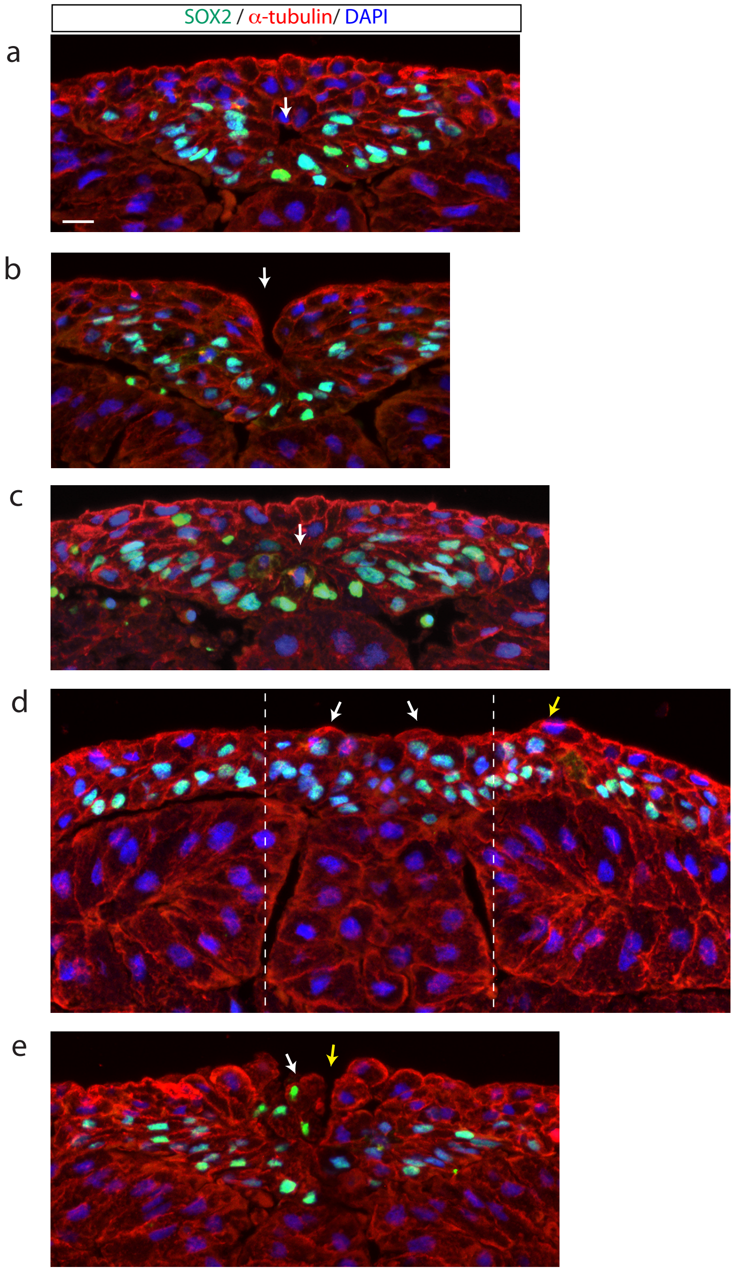

Panel	Neural tissue defect score
a	0
b	1<br>Open neural tube: 1
c	1<br>No lumen: 1
d	3<br>Open neural tube: 1<br>Unfolded neural plate: 1<br>Rounded cells: 1
e	3<br>Open neural tube : 1<br>Neural tissue disruption: 1<br>Rounded cells: 1

Supplementary Fig. 3. Scoring of neural tissue defects in *Xenopus laevis* embryos.
Control and experimental *Xenopus laevis* embryos were allowed to develop until controls reached early neural tube stages (stage 21), when they were fixed and processed for immunostaining. **a**-**e**, Images are examples of immunostained and nuclei-labeled transverse sections. Characteristics of defects and scores are listed on the right. Scale bar, 20 μm. White arrows indicate lumen (**a**), open neural tube (**b**), absence of lumen (**c**), rounded and loosely attached neural cells (**d** and **e**). Yellow arrows indicate neural fold (**d**) and neural tissue disruption (**e**). Dashed lines in (**d**) were traced as perpendicular to the dorsal plane and tangent to the lateral borders of the notochord and used to identify the lack of neural plate folding when neural folds do not fall in between these lines. Text to the right of images indicates the score of neural tissue defects and type of defects identified in each shown example.

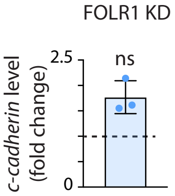
FOLR1 KD
ns
2.5
0
c-cadherin level
(fold change)

Supplementary Fig. 4. FOLR1 knockdown in *Xenopus laevis* embryos does not affect *c-cadherin* transcription.

Two-cell stage embryos were injected with 3.2 pmol Control-morpholino (Control) or FOLR1-splicing-blocking morpholino (FOLR1 KD) per blastomere. Neural plates from injected stage-16 *Xenopus laevis* embryos were dissected and processed for mRNA extraction followed by *in vitro* conversion into cDNA. Samples were subjected to quantitative PCR assays with ODC as normalizing transcript. Graph shows individual and mean±SD fold change in *c-cadherin* transcript level compared to Control samples (1, dashed line), ns: not significant, two-tailed ratio t-test, n=27 neural plates per group, N=3 independent experiments.

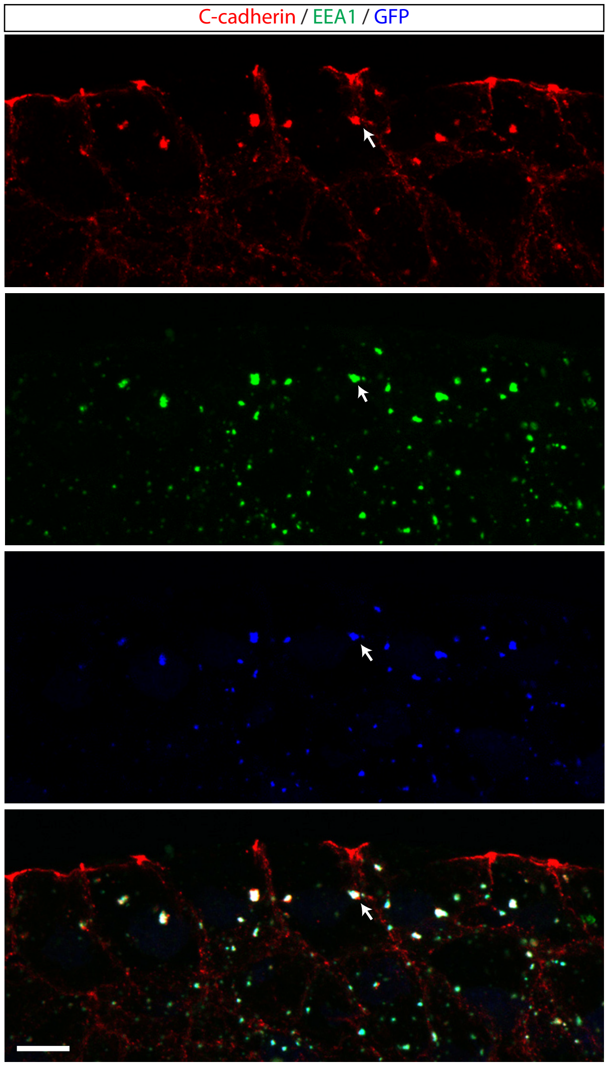
C-cadherin / EEA1 / GFP

Supplementary Fig. 5. Exogenously expressed endocytosis reporter hEEA1-GFP colocalizes with endogenously-expressed C-cadherin in early endosomes.

Two-cell stage *Xenopus laevis* embryos were bilaterally microinjected with hEEA1-GFP mRNA and allowed to develop until they reached early neural plate stages (stage 14-14.5), when they were fixed and processed for immunostaining. Image is a transverse section of the neural plate immunostained for C-cadherin, EEA1 and GFP. Arrow indicates colocalization of C-cadherin, EEA1 and GFP. Scale bar, 10 μm. Similar results were observed in N=3 independent experiments.

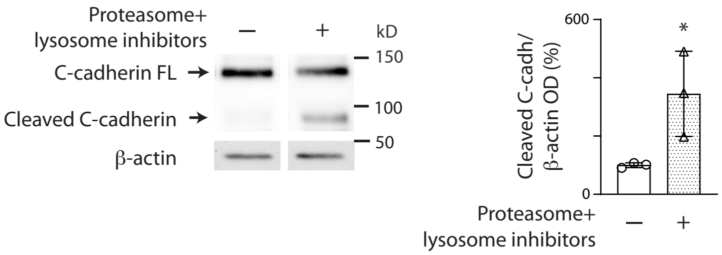

Proteasome+
lysosome inhibitors
−
+
kD
150
100
50
C-cadherin FL
Cleaved C-cadherin
β-actin
Cleaved C-cadh/
β-actin OD (%)
600
0
*
Proteasome+
lysosome inhibitors
−
+

Supplementary Fig. 6. Inhibition of protein degradation in neurulating *Xenopus laevis* embryos results in accumulation of C-cadherin N-terminal fragment in neural plate.

Early neural plate stage (stage 13) embryos were incubated with lysosome and proteasome inhibitors or vehicle for 4-5 h until they reached mid-neural plate stages (stage 16-17) when neural plate was dissected and processed for Western blot assays. Example of Western blot assay immunoprobed for C-cadherin showing full-length and cleaved (~80 kD) forms. Graph shows individual and mean±SD percent of optical density (OD) for C-cadherin cleaved fragment normalized with β-actin protein band OD and compared to controls (100%, dashed line), *$p<0.05$, two-tailed ratio t-test, n=10 neural plates per group, N=3 independent experiments.

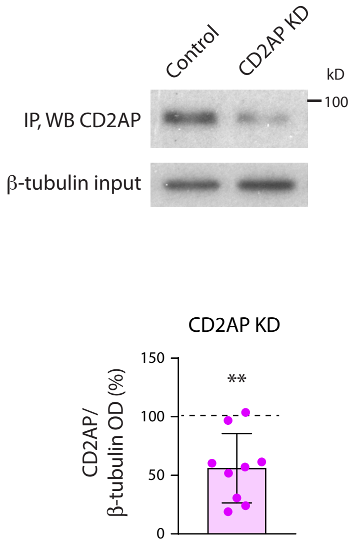
Control
CD2AP KD
kD
100
IP, WB CD2AP
β-tubulin input
CD2AP KD
150
100
50
0
**
CD2AP/
β-tubulin OD (%)

Supplementary Fig. 7. CD2AP knockdown.

Two-cell stage *Xenopus laevis* embryos were bilaterally microinjected with 9.9 pmol Control-morpholino (Control) or CD2AP-morpholino 2 (CD2AP KD) per blastomere and allowed to grow until they reached early neural tube stages (stage 20) when embryos were collected and processed for immunoprecipitation (IP) followed by Western blot (WB) assays. Image is an example of a Western blot assay. Graph shows individual and mean±SD percent of optical density (OD) for CD2AP protein band normalized with β-tubulin protein band OD and compared to controls (100%, dashed line). $^{**}p<0.01$, two-tailed ratio t-test, n=45 embryos, N=3 independent experiments.

Protein name/ UniProt accession #	Replicate	# Peptides identified FOLR1-IP	# Peptides identified Control-IP
**β-catenin Ctnnb1/** A1A5I6	1	4	0
	2	5	0
	3	4	0
**C-cadherin Xb-cadherin/** Q6NTM0	1	1	0
	2	7	0
	3	4	0
**CD2AP/** Q4V7X8	1	2	0
	2	2	0
	3	2	0

Supplementary Table 1. FOLR1-interacting proteins during neural plate stages.
Lysates from neural plate stage *Xenopus laevis* embryos were immunoprecipitated with anti-FOLR1 antibody and immunoprecipitates (IP) were processed for LC-MS/MS assays. Shown are numbers of peptides identified for a selection of FOLR1-interacting proteins in each run.

Full scans of Western blots presented in Suppl. Fig. 1a

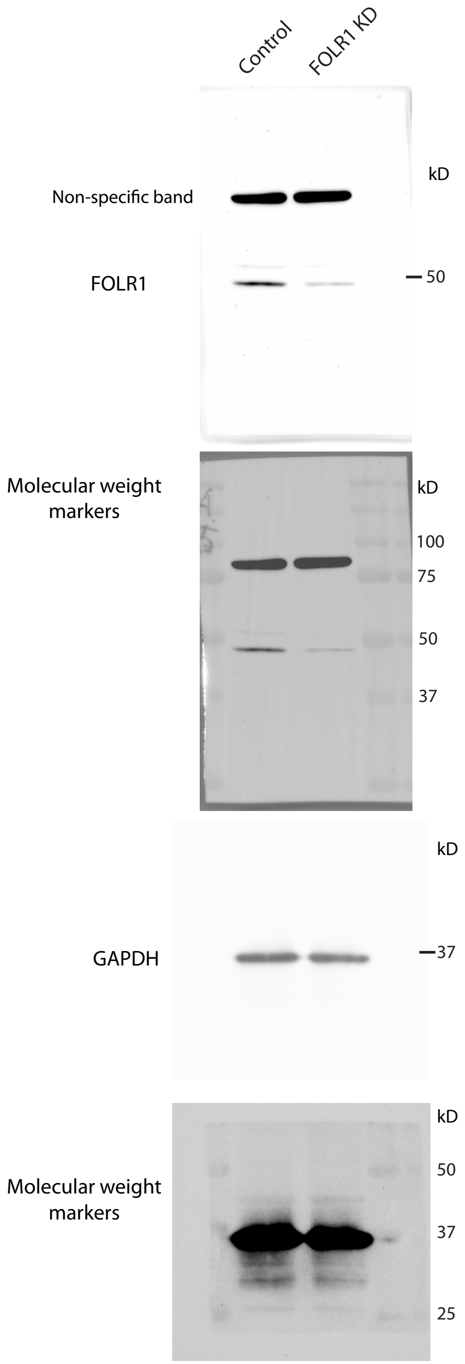

Full scan of gel presented in Suppl. Fig. 1c

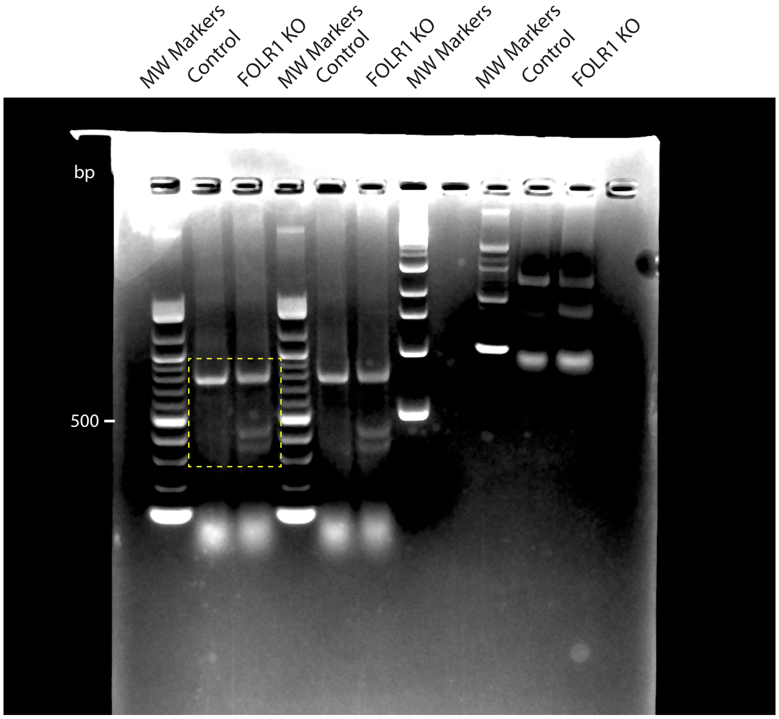

Full scans of Western blots presented in Suppl. Fig. 1d

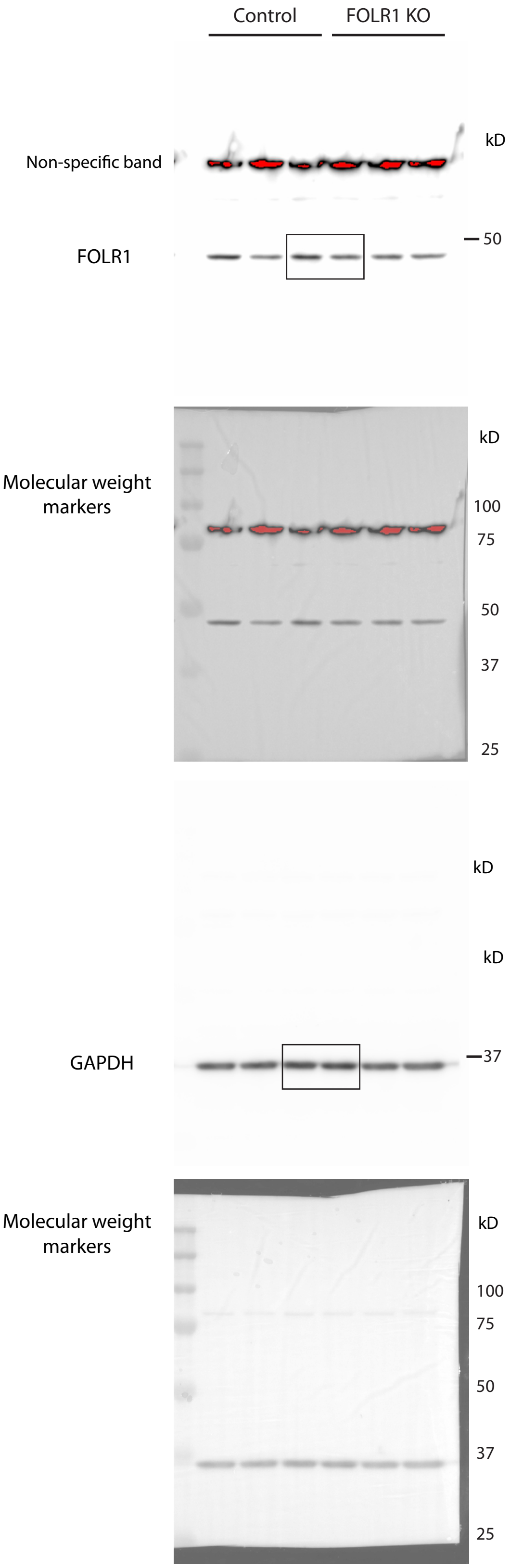

Full scans of Western blots presented in Suppl. Fig. 6

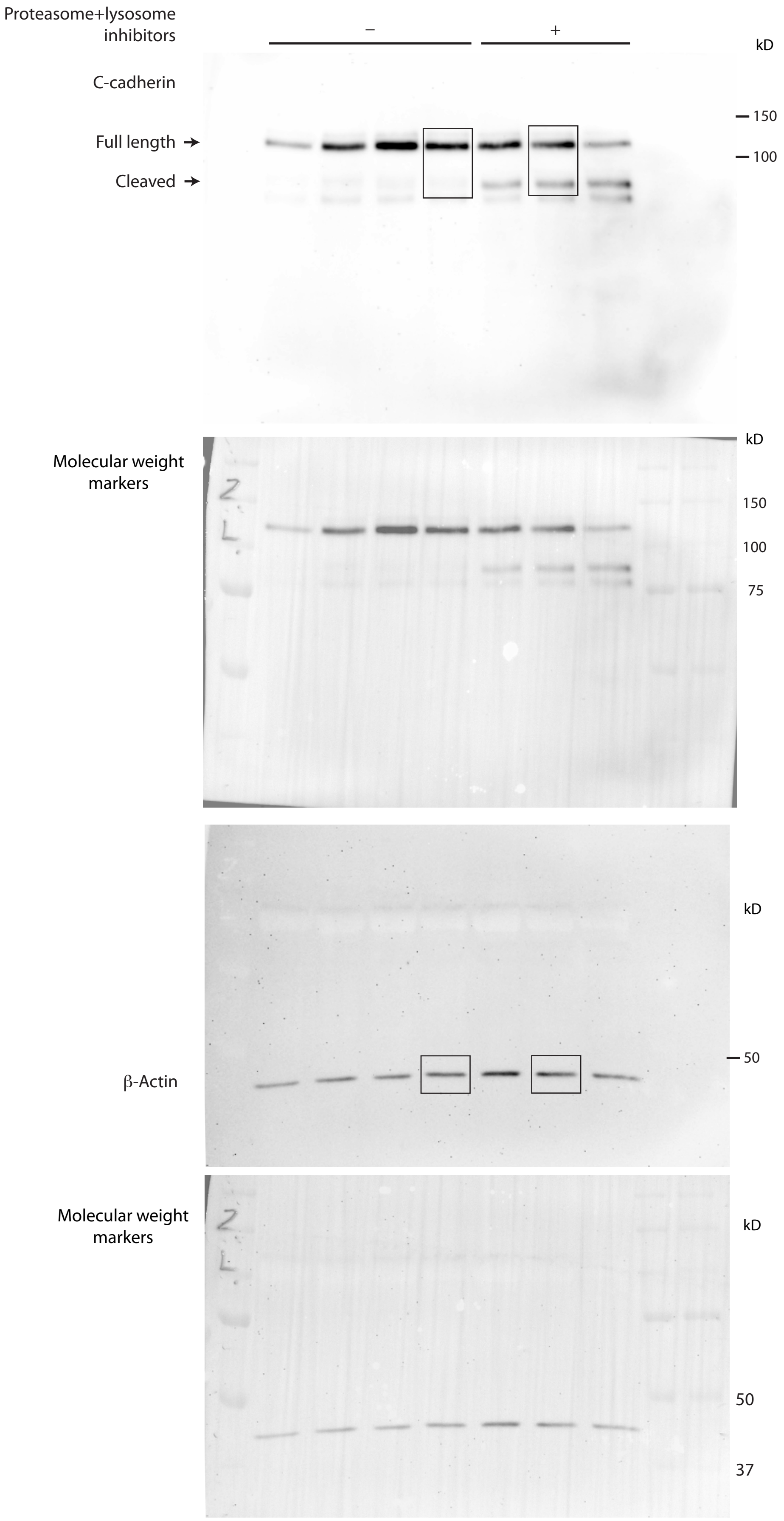

Full scans of Western blots presented in Suppl. Fig. 7

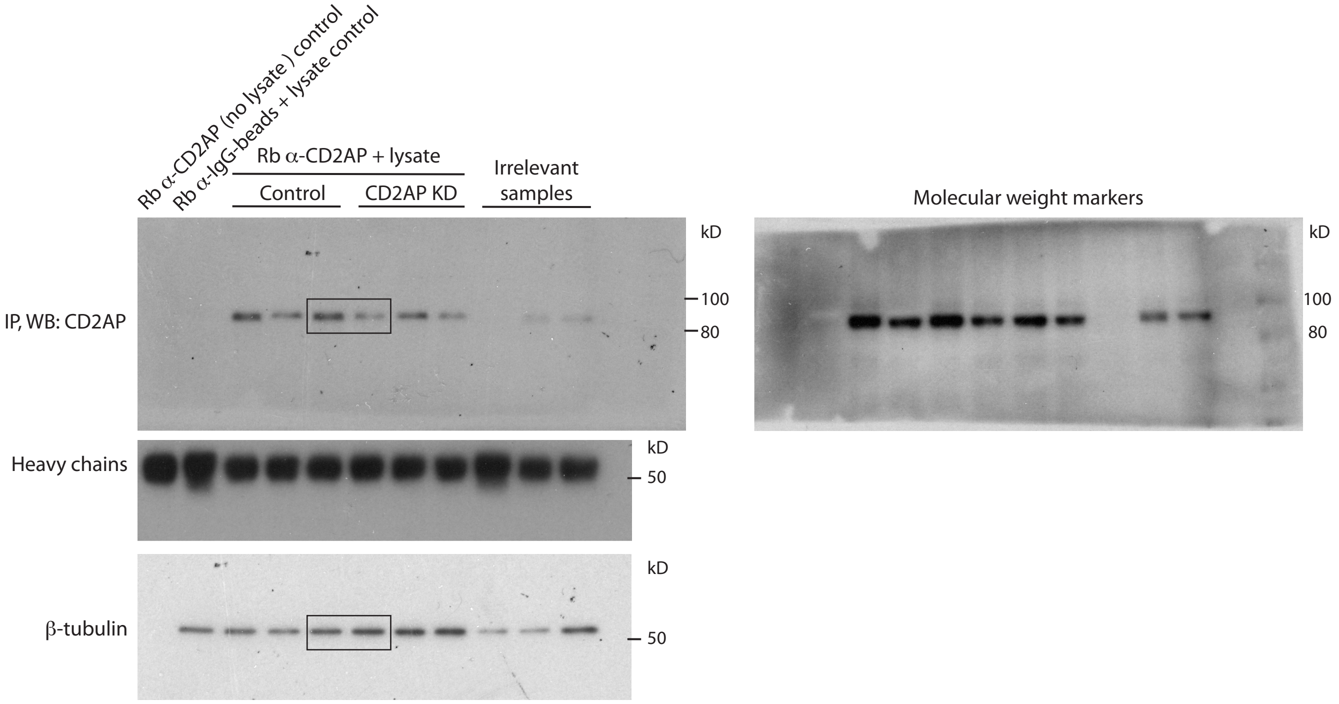

Supplement: Supplementary file 1 — Supplementary Information [file 41467_2024_45775_MOESM1_ESM.pdf]
