# Supplementary material for: Noncanonical function of folate through folate receptor 1 during neural tube formation
Source: Nat Commun. 2024 Feb 22;15:1642. doi: 10.1038/s41467-024-45775-1 (PMC10883926; doi:10.1038/s41467-024-45775-1)
Supplement: Supplementary file 3 — Reporting Summary [file 41467_2024_45775_MOESM3_ESM.pdf]

nature portfolio

Corresponding author(s): Laura Borodinsky

Last updated by author(s): Jan 17, 2024

Reporting Summary

Nature Portfolio wishes to improve the reproducibility of the work that we publish. This form provides structure for consistency and transparency in reporting. For further information on Nature Portfolio policies, see our Editorial Policies and the Editorial Policy Checklist.

Statistics

For all statistical analyses, confirm that the following items are present in the figure legend, table legend, main text, or Methods section.

n/a	Confirmed	
☐	☒	The exact sample size (*n*) for each experimental group/condition, given as a discrete number and unit of measurement
☐	☒	A statement on whether measurements were taken from distinct samples or whether the same sample was measured repeatedly
☐	☒	The statistical test(s) used AND whether they are one- or two-sided<br>*Only common tests should be described solely by name; describe more complex techniques in the Methods section.*
☐	☒	A description of all covariates tested
☐	☒	A description of any assumptions or corrections, such as tests of normality and adjustment for multiple comparisons
☐	☒	A full description of the statistical parameters including central tendency (e.g. means) or other basic estimates (e.g. regression coefficient) AND variation (e.g. standard deviation) or associated estimates of uncertainty (e.g. confidence intervals)
☐	☒	For null hypothesis testing, the test statistic (e.g. *F*, *t*, *r*) with confidence intervals, effect sizes, degrees of freedom and *P* value noted<br>*Give P values as exact values whenever suitable.*
☒	☐	For Bayesian analysis, information on the choice of priors and Markov chain Monte Carlo settings
☒	☐	For hierarchical and complex designs, identification of the appropriate level for tests and full reporting of outcomes
☒	☐	Estimates of effect sizes (e.g. Cohen's *d*, Pearson's *r*), indicating how they were calculated

Our web collection on statistics for biologists contains articles on many of the points above.

Software and code

Policy information about availability of computer code

Data collection	NIS Elements (Nikon Inc.), Image Lab Software (Bio-Rad, Inc.).
Data analysis	NIS Elements (Nikon), Prism 10 (GraphPad), Image Lab Software (BioRad)

For manuscripts utilizing custom algorithms or software that are central to the research but not yet described in published literature, software must be made available to editors and reviewers. We strongly encourage code deposition in a community repository (e.g. GitHub). See the Nature Portfolio guidelines for submitting code & software for further information.

Data

Policy information about availability of data

All manuscripts must include a data availability statement. This statement should provide the following information, where applicable:

- Accession codes, unique identifiers, or web links for publicly available datasets
- A description of any restrictions on data availability
- For clinical datasets or third party data, please ensure that the statement adheres to our policy

All data supporting the findings of this study are available within the paper and its Supplementary Information. Source data are provided as Source Data files with this paper. The mass spectrometry proteomics data have been deposited to the ProteomeXchange Consortium via the PRIDE 63 partner repository with the dataset identifier PXD048476 and 10.6019/PXD048476. Project Name: FOLR1-interacting proteins in neurulating Xenopus laevis embryos. Project accession: PXD048476. Project DOI: 10.6019/PXD048476. Reviewer account details: Username: reviewer_pxd048476@ebi.ac.uk. Password: 0DFthm7n.

Research involving human participants, their data, or biological material

Policy information about studies with human participants or human data. See also policy information about sex, gender (identity/presentation), and sexual orientation and race, ethnicity and racism.

Reporting on sex and gender	N/A
Reporting on race, ethnicity, or other socially relevant groupings	N/A
Population characteristics	N/A
Recruitment	N/A
Ethics oversight	N/A

Note that full information on the approval of the study protocol must also be provided in the manuscript.

Field-specific reporting

Please select the one below that is the best fit for your research. If you are not sure, read the appropriate sections before making your selection.

☒ Life sciences ☐ Behavioural & social sciences ☐ Ecological, evolutionary & environmental sciences

For a reference copy of the document with all sections, see nature.com/documents/nr-reporting-summary-flat.pdf

Life sciences study design

All studies must disclose on these points even when the disclosure is negative.

Sample size	Power analysis and pilot experiments were used to determine sample size for all sets of data.
Data exclusions	There was no data exclusion.
Replication	Number of samples for each experiment was determined by pilot experiments and power analysis. Experiments were replicated at least 3 times. No data were excluded from the analysis.
Randomization	Independent experiments using different batches of embryos or neural organoids constituted the N of experiments included in statistical analysis. Different groups were randomly sorted from the original batch of samples to have equal representation of unaccounted variables in control and experimental groups.
Blinding	Whenever possible, experimenters were blinded to the type of sample being analyzed by coding samples in a way that hid their identity. Blinding was not implemented in gel analysis because this was done directly by the gel developer software. Thus, the analysis was unbiased and did not require blinding from the experimenter.

Reporting for specific materials, systems and methods

We require information from authors about some types of materials, experimental systems and methods used in many studies. Here, indicate whether each material, system or method listed is relevant to your study. If you are not sure if a list item applies to your research, read the appropriate section before selecting a response.

Materials & experimental systems

n/a	Involved in the study	
☐	☒	Antibodies
☐	☒	Eukaryotic cell lines
☒	☐	Palaeontology and archaeology
☐	☒	Animals and other organisms
☒	☐	Clinical data
☒	☐	Dual use research of concern
☒	☐	Plants

Methods

n/a	Involved in the study	
☒	☐	ChIP-seq
☒	☐	Flow cytometry
☒	☐	MRI-based neuroimaging

Antibodies

Antibodies used	Anti-FOLR1, 1:250 (Bioworld Technologies, cat. # BS386); anti-SOX2, 1:200 (R&D Systems, cat. # AF2018); anti-pan-cadherin, 1:300 (Abcam, cat. # ab22744); anti-α-catenin, 1:250 (Invitrogen, cat. # 13-9700); anti-FOLR1, 1:500 in 5% BSA in TBST (Bioworld Technology, cat. # BS3861); anti-alpha-tubulin, 1:500 (Abcam, cat. # ab15246); anti-phospho-tyr-4,8,10 CD2AP, 1:500 (Rockland, cat.

600-401-J96), anti-phospho-tyr-674 c-Cbl, 1:500 (MyBioSource, cat. # MBS820886), anti-C-cadherin, 1:100 (Developmental Studies Hybridoma Bank, cat. # 6B6), anti-EEA1, 1:1000 (Origene, cat. # AB0006-200), anti-ubiquitin, 1:500 (Stress Marq, cat. # SPC-119B), anti-Rab7, 1:500 (Cell Signaling, cat. # 9367), anti-LAMP1, 1:500 (Abcam, cat. # ab24170), anti-GFP, 1:750 (Abcam, cat. # ab13970), anti-mCherry, 1:750 (Biorbyt, cat. # orb11618); anti-Xenopus FOLR1 and anti-Xenopus CD2AP, costum-made, Genescript; Alexa Fluor secondary antibodies: anti-mouse 647 donkey, 1:300 (Invitrogen, cat. # A31571), anti-rabbit 488 donkey (Invitrogen, cat. # A21206) and anti-goat 596 donkey (Invitrogen, cat. # A11057); HRP-conjugated secondary antibodies, 1:7,500 (Jackson ImmunoResearch, cat. # 711-035-152)

Validation

Validation of antibodies was performed by knockdown of the target protein followed by Western blot assays and by confirming reported subcellular localization apparent in our samples. Additional controls included using irrelevant IgGs for co-IP experiments and incubation with secondary antibodies only for immunohistochemistry assays.When possible, commercially validated antibodies were used. Validation of those antibodies was verified in published studies, homology analysis of proteins and antigens across species and data provided by vendors.

Eukaryotic cell lines

Policy information about cell lines and Sex and Gender in Research

Cell line source(s)

Human wild type induced pluripotent stem cells (hiPSCs; WTC-11 hiPSC line, Allen Institute, GM25256, Coriell Institute for Medical Research)

Authentication

Authentication provided by the Allen Institute through karyotypic analysis before cell line submission to CCR. Cell line was evaluated for viability surface antigen expression and alkaline phosphatase activity. Pluripotency was assessed via embryoid body (EB) formation. Steady-state mRNA expression patterns of undifferentiated iPSC and EBs were determined via real-time PCR.

Mycoplasma contamination

Negative

Commonly misidentified lines
(See ICLAC register)

No commonly misidentified lines were associated with the utilized cell line.

Animals and other research organisms

Policy information about studies involving animals; ARRIVE guidelines recommended for reporting animal research, and Sex and Gender in Research

Laboratory animals

Adult male and female wild type Xenopus laevis frogs

Wild animals

The study did not involve wild animals

Reporting on sex

Developmental stage too early to determine/or being sex a relevant variable.

Field-collected samples

The study did not involve samples collected from the field.

Ethics oversight

The Institutional Animal Care and Use Committee (IACUC) approved and oversaw the animal handling and care procedures utilized in this study. IACUC follows the guidelines established by the Animal Welfare Act and the Public Health Service Policy on Humane Care and Use of Laboratory Animals.

Note that full information on the approval of the study protocol must also be provided in the manuscript.

Plants

Seed stocks

N/A

Novel plant genotypes

N/A

Authentication

N/A
