# Supplementary figures and images for: Noncanonical function of folate through folate receptor 1 during neural tube formation
Source: Nat Commun. 2024 Feb 22;15:1642. doi: 10.1038/s41467-024-45775-1 (PMC10883926; doi:10.1038/s41467-024-45775-1)

Full scans of Western blots presented in Figure 4a

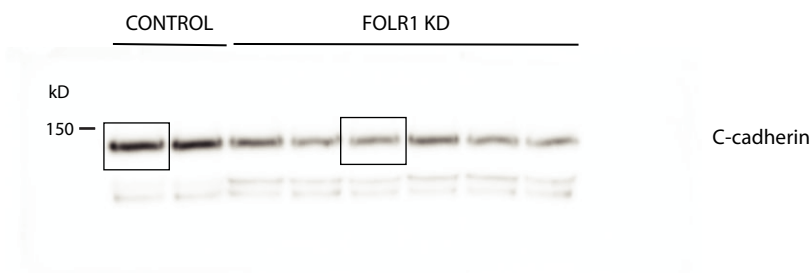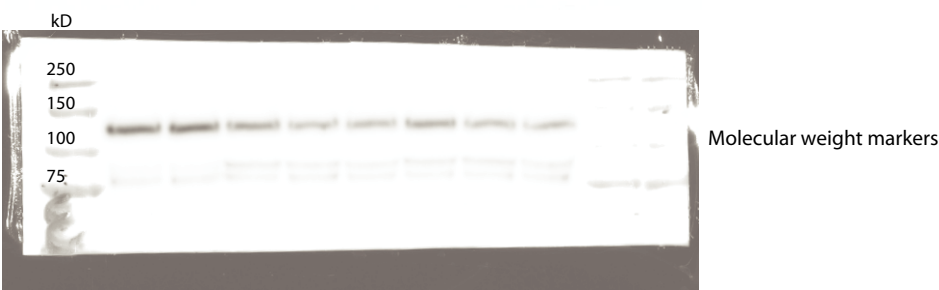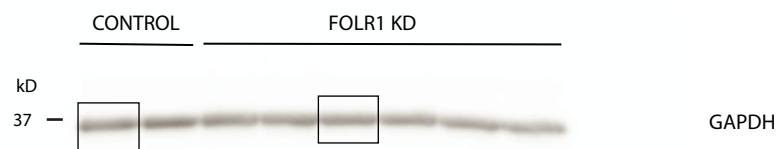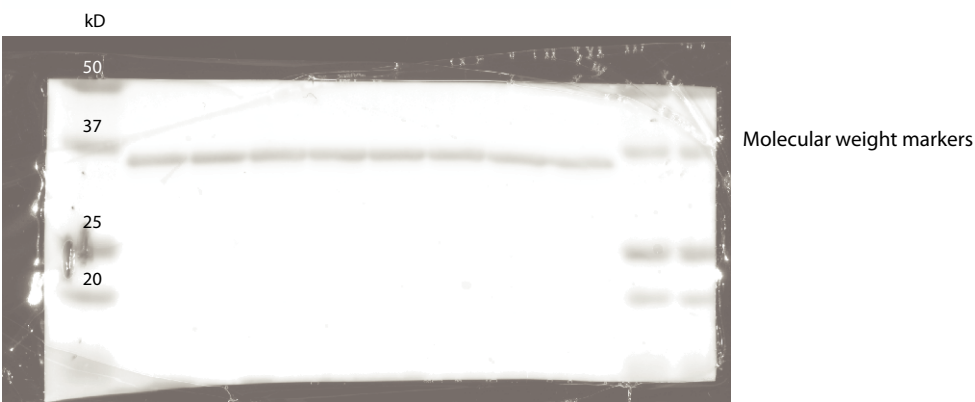

Supplement: Supplementary file 4 — Source Data [file 41467_2024_45775_MOESM4_ESM.zip › Fig 4a-Source Data.pdf]

Full scans of Western blots presented in Fig. 5a

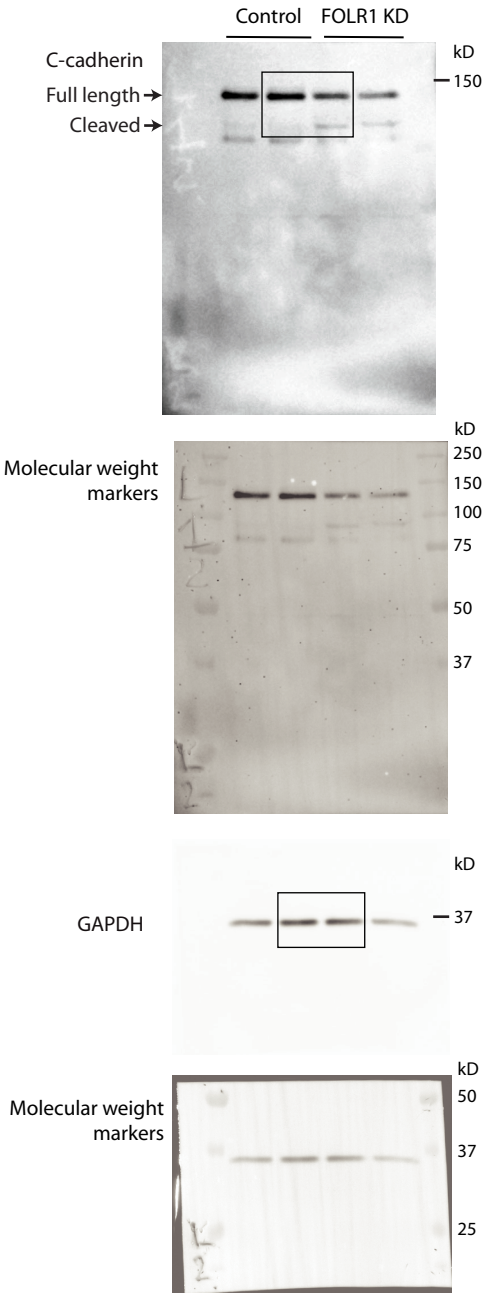

Full scans of Western blots presented in Fig. 5b

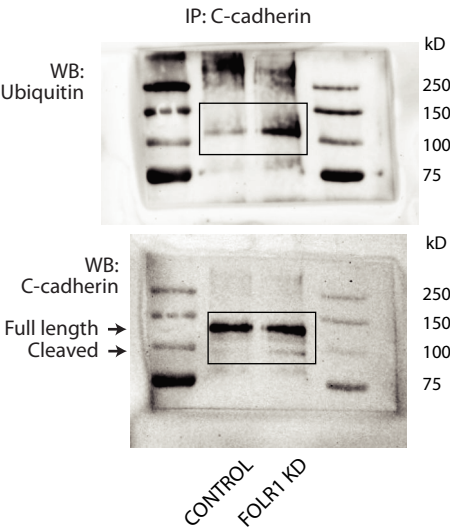

Supplement: Supplementary file 4 — Source Data [file 41467_2024_45775_MOESM4_ESM.zip › Fig 5-Source Data.pdf]

Full scans of Western blots presented in Fig. 6a and full scans of additional control blots

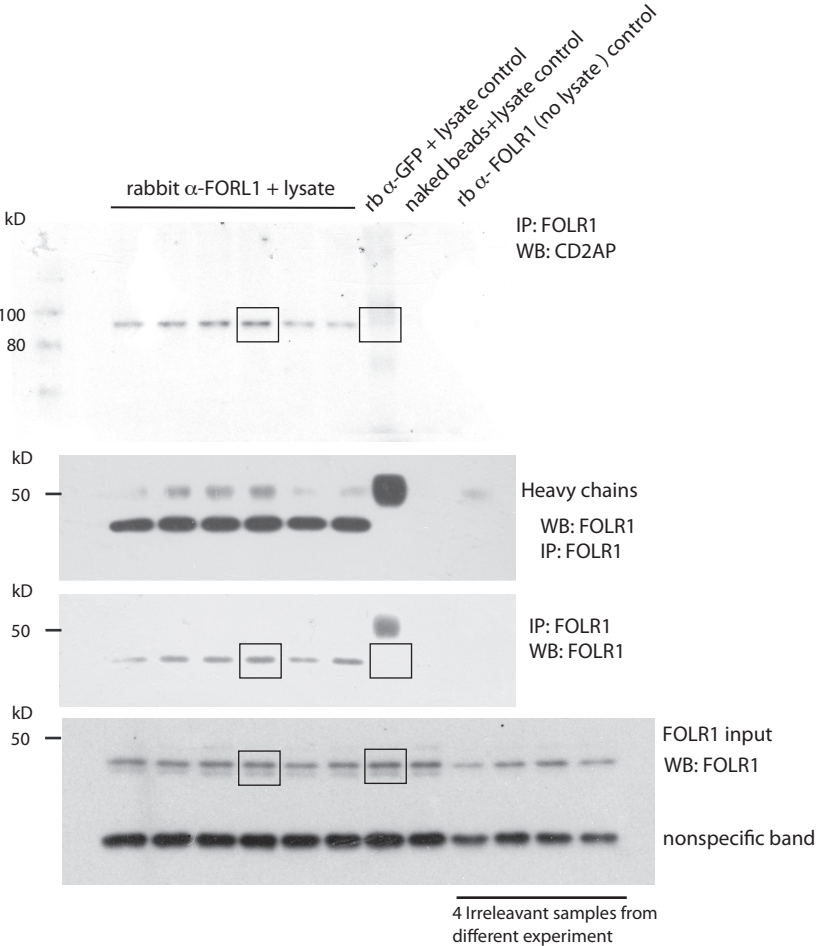

Supplement: Supplementary file 4 — Source Data [file 41467_2024_45775_MOESM4_ESM.zip › Fig 6-Source Data.pdf]

Full scans of Western blots presented in Fig. 7b

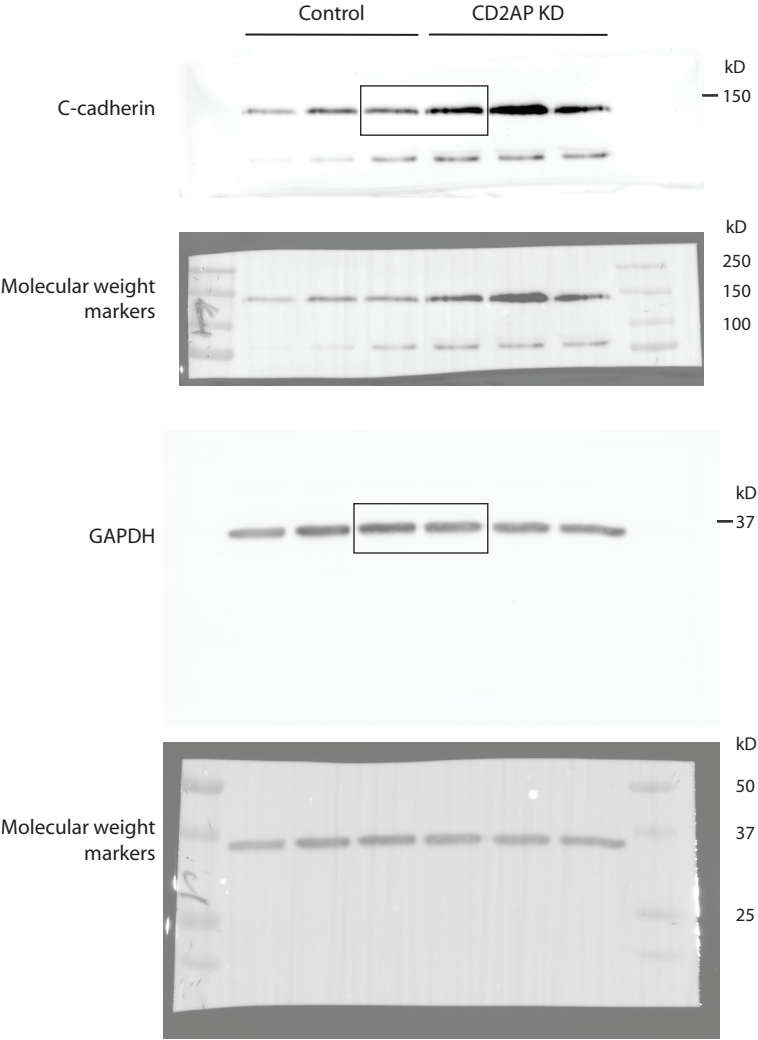

Supplement: Supplementary file 4 — Source Data [file 41467_2024_45775_MOESM4_ESM.zip › Fig 7-Source Data.pdf]

Full scans of Western blots presented in Fig. 8a

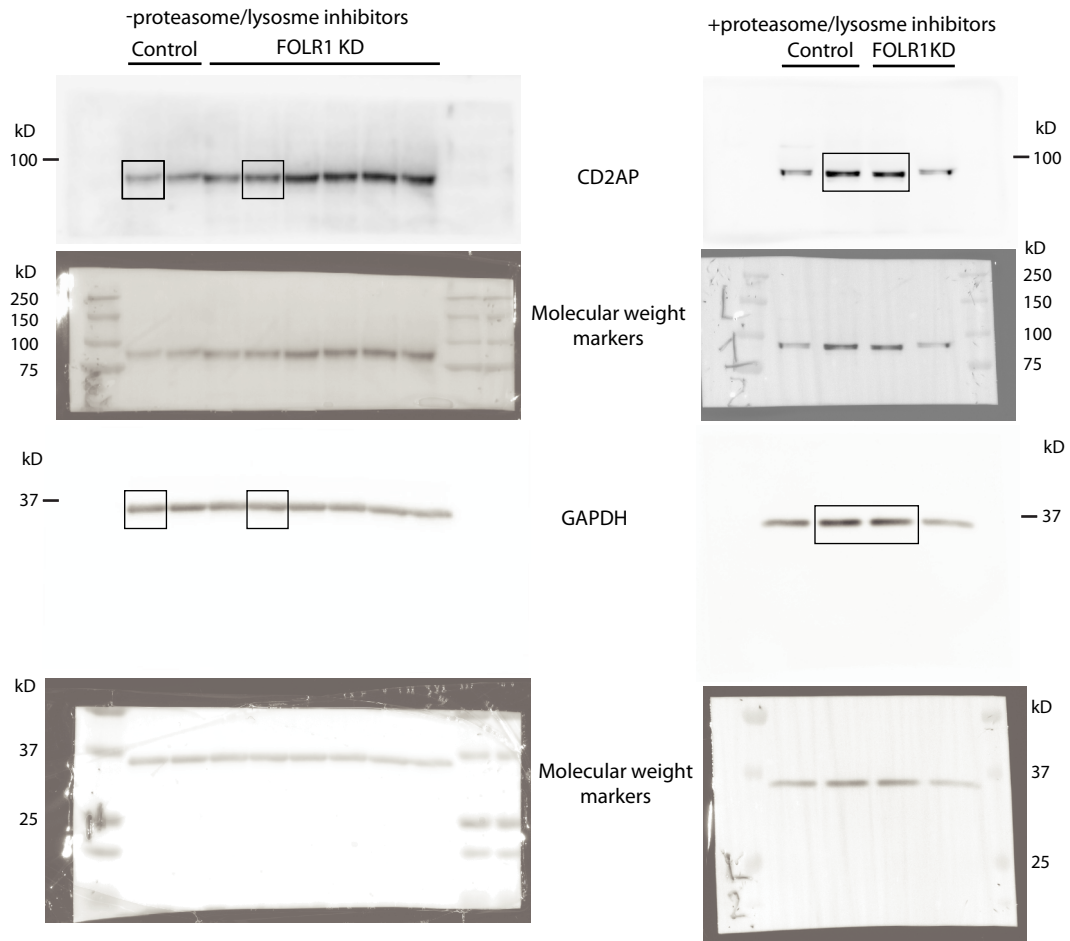

Supplement: Supplementary file 4 — Source Data [file 41467_2024_45775_MOESM4_ESM.zip › Fig 8a-Source Data.pdf]

Full scans of Western blots presented in Fig. 8b

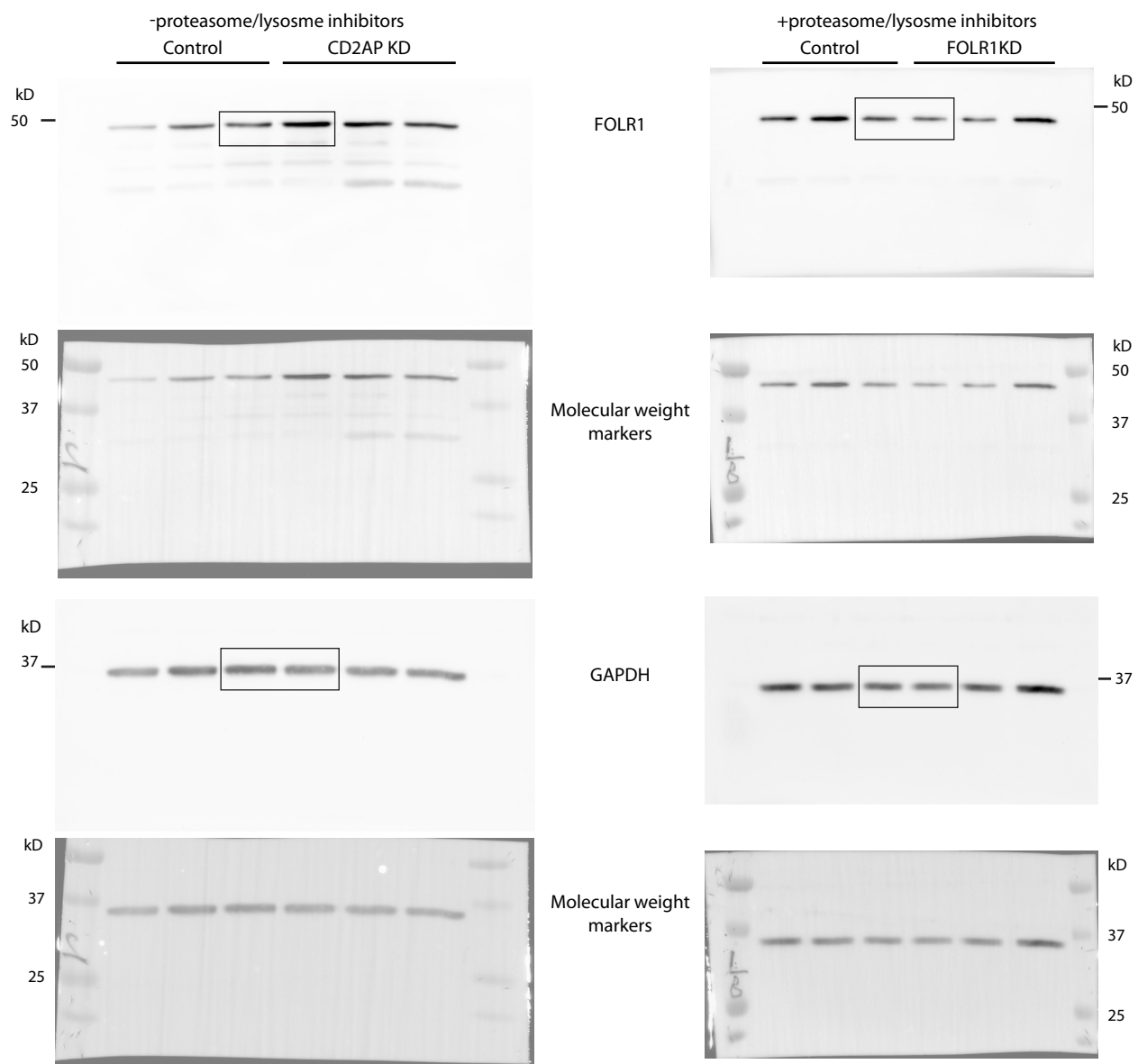

Supplement: Supplementary file 4 — Source Data [file 41467_2024_45775_MOESM4_ESM.zip › Fig 8b-Source Data.pdf]
